# Ultrasonographic assessment of bypass capacity after revascularization surgery in moyamoya disease: a systematic review and single-arm meta-analysis

**DOI:** 10.1007/s00701-025-06658-6

**Published:** 2025-09-10

**Authors:** Natalia Anna Koc, Maurycy Rakowski, Samuel D. Pettersson, Paulina Skrzypkowska, Tomasz Szmuda, Piotr Zieliński

**Affiliations:** 1 Department of Neurosurgery, Medical University of Gdańsk, Gdańsk, Poland; 2 Neurosurgical Service, Beth Israel Deaconess Medical Center, Harvard Medical School, Boston, MA USA

**Keywords:** Moyamoya disease, Cerebral bypass, Revascularization surgery, Ultrasonography

## Abstract

**Purpose:**

Moyamoya disease (MMD) is a chronic cerebrovascular disorder characterized by progressive arterial stenosis and fragile collateral formation, elevating stroke risk. Revascularization is the standard treatment, yet up to 27% of patients experience ischemic events within a year due to bypass insufficiency. While digital subtraction angiography (DSA) remains the gold standard for assessing bypass function, it is invasive and time-consuming. This study evaluates ultrasonography (US) as a noninvasive, cost-effective tool to assess bypass capacity post-revascularization in MMD.

**Methods:**

A systematic search was conducted following PRISMA guidelines. PubMed, Web of Science, and Scopus were searched for studies reporting US parameters with control imaging confirming bypass capacity. Study quality was assessed using the Newcastle–Ottawa Scale. Mean difference (MD) values were calculated using random-effects models. High bypass capacity was defined as good patency or favorable collateral development.

**Results:**

Eight cohort studies comprising 264 MMD patients and 301 operated hemispheres were included, with 180 demonstrating high bypass capacity. Within two weeks post-surgery, increased superficial temporal artery (STA) peak systolic velocity (PSV, MD = 28.26, *p* < 0.0001), mean flow velocity (MFV, MD = 22.97, *p* = 0.03), end-diastolic velocity (EDV, MD = 33.45, *p* < 0.0001), and decreased resistance index (RI, MD = –0.09, *p* = 0.006) were predictive. External carotid artery (ECA) EDV (MD = 13.92, *p* = 0.04) was also significant. At 3–6 months, elevated EDV in both STA (MD = 8.13, *p* = 0.006) and ECA (MD = 8.71, *p* = 0.0002) remained predictive. In the indirect subgroup, lower anterior cerebral artery (ACA) MFV within 0–3 months predicted favorable outcomes (MD = –64.98, *p* = 0.001).

**Conclusions:**

Changes in STA and ECA US parameters measured following revascularization surgery predict high bypass capacity. Decreased ACA MFV suggests effective revascularization after indirect surgery. Ultrasound modality offers a valuable, noninvasive tool for postoperative assessment in MMD.

**Supplementary Information:**

The online version contains supplementary material available at 10.1007/s00701-025-06658-6.

## Introduction

Moyamoya disease (MMD) is a chronic, progressive occlusive cerebrovascular disorder of uncertain pathophysiology, primarily affecting the terminal segments of the internal carotid arteries (ICAs) and their proximal branches. Histopathologically, it involves intimal hyperplasia and arterial wall fibrosis, leading to progressive stenosis or occlusion. These changes trigger fragile collateral networks that attempt to compensate for hypoperfusion but are prone to rupture, causing ischemia or hemorrhage with high morbidity and mortality in both children and adults [4]. Given that unstable MMD poses a significant risk for peri- and postoperative ischemic complications, early recognition of disease severity is essential [27].

Revascularization surgery is an effective treatment option for restoring cerebral blood flow (CBF) and preventing stroke in MMD. Common techniques include direct surgery, creating an extracranial-intracranial (EC-IC) superficial temporal artery-middle cerebral artery (STA-MCA) anastomosis, and indirect methods such as encephalo-duro-arterio-synangiosis (EDAS), encephalo-myo-synangiosis (EMS), encephalo-arterio-synangiosis (EAS), encephalo-duro-synangiosis (EDS), encephalo-myo-arterio-synangiosis (EMAS), or combinations of the above [16, 21]. The increase in postoperative collateral development is related to favorable patient prognosis, while good bypass patency ensures the appropriate blood flow, preventing postoperative ischemic events (PIEs). The benefit of revascularization mainly depends on bypass function, which may be compromised by primary or secondary bypass insufficiency [21].

The success of a revascularization surgery is traditionally assessed using digital subtraction angiography (DSA), the gold standard for MMD assessment [17]. However, DSA’s invasiveness and radiation exposure limit its routine use for longitudinal surveillance [21]. As alternatives, magnetic resonance angiography (MRA) and computed tomography angiography (CTA) have been utilized to evaluate bypass patency. While MRA offers radiation-free imaging, it may be less sensitive for detecting small-caliber bypass vessels or early-stage neovascularization. CTA, although accurate and rapid, involves radiation and contrast exposure [6]. Ultrasonography (US) presents a promising non-invasive, cost-effective, and repeatable alternative to assess bypass function. Prior studies suggest ultrasound parameters may reflect revascularization success, but its clinical value postoperatively yet remains unclear [12, 22, 23]. Therefore, the aim of our study was to systematically evaluate ultrasound parameters as potential predictors of high bypass capacity following revascularization surgery in patients with MMD.

## Materials and methods

### Search strategy

The screening process was performed according to the guidelines of Preferred Reporting Items for Systematic Reviews and Meta-Analyses (PRISMA). Studies were retrieved from PubMed, Web of Science, and Scopus databases from inception to November 6, 2024 using the following keywords: (“moyamoya disease” OR “moyamoya syndrome” OR “MMD”) AND (“revascularization” OR “bypass surgery” OR “STA-MCA bypass” OR “indirect surgery” OR “direct surgery”) AND (“ultrasonography” OR “ultrasound” OR “transcranial doppler” OR “TCD” OR “TCCS”). Two authors (N.A.K. and M.R.) independently conducted the database search. The study protocol was registered via PROSPERO (registration no. CRD42025634331, https://www.crd.york.ac.uk/PROSPERO/).

### Study selection

The inclusion criteria were based on PICOTT (Table 1). Each study had to (1) report patients with MMD undergoing bypass revascularization surgery, (2) report parameters of the USS, (3) report a control group of non-USS imaging confirming the patency and function of the bypass, and (4) have a sample size of at least 10 patients. Two authors (N.A.K. and M.R.) independently screened the article titles and abstracts using Rayyan (https://www.rayyan.ai/). The studies that passed the initial screening were reassessed in a full-text screening phase. The studies were required to provide extractable data. All studies that lacked a control group of patients with non-US imaging confirming bypass status were excluded.

**Table 1:** PICOTT criteria used in the study

PICOTT	Description
P	Individuals with MMD treated with cerebral revascularization surgery
I	US with adjunctive MRA, CTA or DSA as reference
C	No control group
O	Ultrasonographic parameters
T	Diagnostic, observational
T	No restriction

### Data extraction

Data extraction focused on postoperative ultrasound parameters associated with high-capacity bypass function following direct, indirect or combined cerebral revascularization surgery. The following data were also extracted: (1) study design, (2) number of patients, (3) patient demographics, (4) number of operated hemispheres, (5) surgery procedure, (6) ultrasound method, (7) control imaging modality, (8) postoperative time points of measurement, (9) assessed arteries. Bypass capacity was categorized as high if there was good bypass patency, lack of postoperative ischemic events (PIEs), or good collateral formation (A or B grade according to the Matsushima criteria), and as low if poor patency, PIEs, or insufficient collateral development was observed (C or D according to the Matsushima criteria) [14]. For studies presenting data solely in graphical form, numerical values were extracted using the online version of PlotDigitizer (https://plotdigitizer.com).

### Quality assessment

The quality of included studies was assessed using the Newcastle–Ottawa Scale (NOS). The analyzed studies could earn a maximum of 4 points in the selection category, 2 points in the comparability category, and 3 points in the outcome category. A total score ≥ 7 indicated high quality, 6–4 moderate quality, and ≤ 3 low quality. The quality assessment was performed by one author (M.R.).

### Statistical analysis

Quantitative analysis was performed on the number of operated hemispheres and associated US parameters reflecting bypass capacity. Random effect models were utilized, and continuous outcomes were synthesized by calculating mean differences (MD) with corresponding 95% confidence intervals (CI). Statistical heterogeneity was assessed using the I^2^ statistic. Publication bias was assessed by visual inspection of funnel plot asymmetry. Statistical significance was defined as a *p*-value < 0.05. Analysis was performed using Cochrane RevMan (Review Manager) version 9.0.0.

## Results

### Search results

**Fig. 1:**
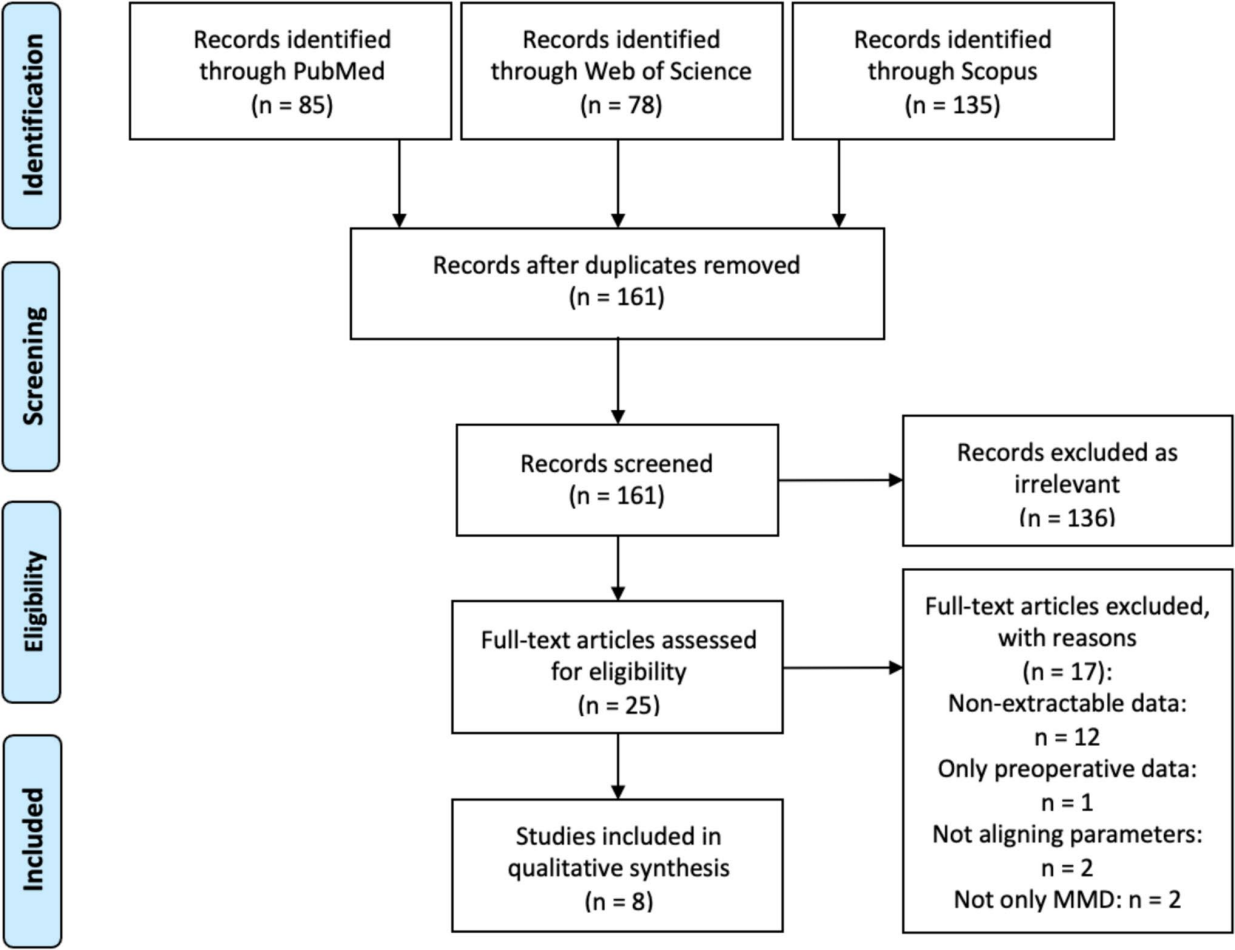
PRISMA diagram showing selection process of the studies. Data added to the template: Moher D, Liberati A, Tetzlaff J, Altman DG, The PRISMA Group (2009). Preferred Reporting Items for Systematic Reviews and Meta-Analyses: The PRISMA Statement. PLoS Med 6(7): e1000097. 10.1371/journal.pmed1000097

As shown in Fig. 1, a comprehensive search of PubMed, Web of Science, and Scopus databases identified 298 potentially eligible studies. After removing duplicates and screening titles and abstracts, 25 full-text articles were assessed for eligibility. Studies were excluded if they: (1) did not allow for data extraction, (2) included only preoperative data, (3) reported non-overlapping US parameters unsuitable for analysis, or (4) included not only MMD patients. Ultimately, 8 studies met the inclusion criteria; of these, 7 were prospective and 1 retrospective. All included studies were rated as high quality according to the Newcastle–Ottawa Scale (Supplementary Table 1).

### Study characteristics

Eight cohort studies encompassing a total of 264 patients with MMD undergoing direct, indirect, or combined cerebral revascularization surgery were included. The characteristics of all included studies are presented in Table 2. A cumulative total of 301 hemispheres were operated on, with 180 hemispheres classified as high-capacity bypass. Regarding surgical techniques, 96 patients underwent direct revascularization, 110 patients received indirect procedures, and 120 patients were treated with a combined approach. To evaluate changes in bypass flow dynamics, we defined three postoperative intervals of interest: within 2 weeks and between 3–6 months from surgery. Intracranial arteries were assessed at the interval within 3 months post-surgery. Ultrasound assessments across studies included the following hemodynamic parameters: peak systolic velocity (PSV, cm/s), end diastolic velocity (EDV, cm/s), resistance index (RI), pulsatility index (PI), and mean flow velocity (MFV, ml/min). These measurements were assessed for the following arteries involved in cerebral perfusion: internal carotid artery (ICA), external carotid artery (ECA), superficial temporal artery (STA), anterior cerebral artery (ACA), middle cerebral artery (MCA), and posterior cerebral artery (PCA).

**Table 2:** Characteristics of all included studies

Author	Year	Country	Study design	No. of patients	Age groups	Mean age	% Female	No. of hemispheres	Surgery method	Ultrasound technique	Reference imaging	Postoperative measurements time points	Arteries assessed
Yeh et al. [26]	2017	Taiwan	Prospective	21	Adults and Children	17.0 ± 10.2	61.9%	24	Indirect (EDAS, EMS, EPS or multiple craniotomies)	Color-coded duplex ultrasound using a linear-array transducer (11–3 MHz) for extracranial arteries (carotid Doppler) and a phased-array probe (5–1 MHz) for intracranial arteries (TCCS)	DSA	Within 0–4 days (mean 1.8 ± 1.3 days)	ICA, ECA, STA, MCA, PCA, ACA
Wang et al. [21]	2022	Japan	Prospective	49	Adults	37.77 ± 7.42	73.5%	49	STA-MCA bypass	Color-coded duplex ultrasound using a 9–3 MHz linear-array transducer for STA and a 12–5 MHz linear-array transducer for ICA, ECA, and VA	DSA	Between 3—6 months	Good vs. poor: STA
Ogawa et al. [15]	2017	Japan	Prospective	21	Adults	42.0 ± 13.9	81.0%	30	Indirect (EDAS)	Color-coded Doppler ultrasonography	DSA	At 2 weeks, 3 months, and 12 months	STA
Matsuo et al. [13]	2021	Japan	Retrospective	28	Adults	40.8 ± 2.4	NR	31	Combined (direct and indirect)	Carotid ultrasound using color-coded systems (Aloka Prosound Alpha 6/7; Hitachi Aloka Medical, Tokyo, Japan) with a 4–13 MHz broadband linear-array transducer	Intraoperative doppler sonography or indocyanine green videoangiography, and postoperative CTA or MRA	At 2 weeks, at 3, 12, and 24 months	ECA, ICA, CCA
Chen et al.^a^ [2]	2023	China	Prospective	52	Adults and Children	39.9 ± 14.3	51.9%	54	Combined (STA-MCA bypass combined with EDMS)	Duplex ultrasonography	DSA	At day 1, day 7, 3 months, and 6 months	STA
Yeh et al. [24]	2024	Taiwan	Prospective	36	Adults and Children	23.0 ± 18.5	52.8%	56	Indirect (EDAS, EPS, EMS or a combination of these)	Color-coded ultrasound system (iE33; Philips Medical Systems, Cambridge, MA, USA) with a 3–11 MHz linear-array transducer for extracranial arteries and a 1–5 MHz phased-array probe for intracranial arteries (with angle correction)	DSA	At 1 and 3 months after surgery	ECA, STA, OA, ACA, MCA, PCA
Wu et al. [22]	2011	China	Prospective	26	Adults	31.0	50.0%	22	STA-MCA bypass	Color Doppler ultrasound system (GE-LOGIQ9; GE Healthcare, London, UK) with a 7L transducer (2.5–7.0 MHz bandwidth)	DSA	At 1 week and 3 months (22 cases)	STA
Chen et al.^b^ [1]	2023	China	Prospective	35	Adults	47.06 ± 9.60	42.9%	35	Combined (direct and indirect)	TCCS using a 4 MHz pulsed Doppler system (DWL, Germany)	CTA	Within 9 days (mean 5 days)	STA

*ACA* anterior cerebral artery, *CCA* common carotid artery, *CTA* computed tomography, *DSA* digital subtraction angiography, *EDAS* encephalo-duro-arterio-synangiosis, *EDMS* encephalo-duro-myo-synangiosis, *ECA* external carotid artery, *EMS* encephalo-myo-synangiosis, *EPS* encephalo-periosteal-synangiosis, *ICA* internal carotid artery, *MCA* middle cerebral artery, *MRA* magnetic resonance angiography, *NR* not reported, *OA* occipital artery, *PCA* posterior cerebral artery, *STA* superficial temporal artery, *TCCS* transcranial color-coded soography, *VA* vertebral artery

### Publication bias

The funnel plot displayed an asymmetrical distribution of the included studies between the MD and the standard error, which indicates the presence of publication bias (Supplementary Fig. 1).

### Predictors of high bypass capacity

**Fig. 2:**
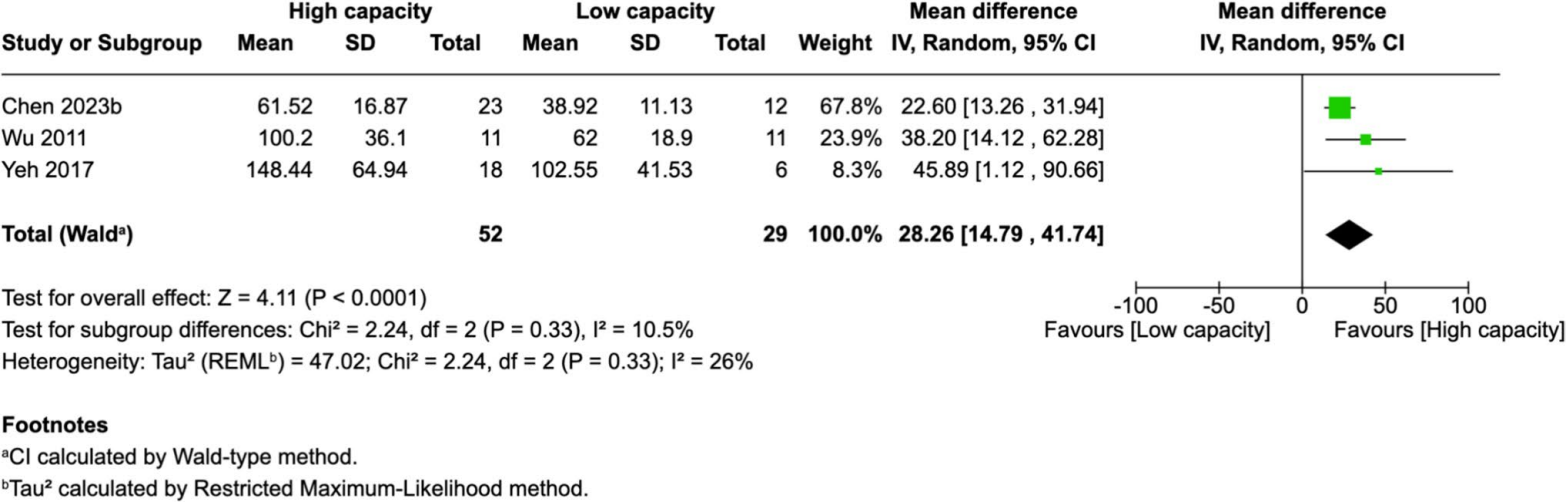
Forest plot showing that higher STA PSV is a significant predictor of high bypass capacity within a timeframe of ≤ 2 weeks

**Fig. 3:**
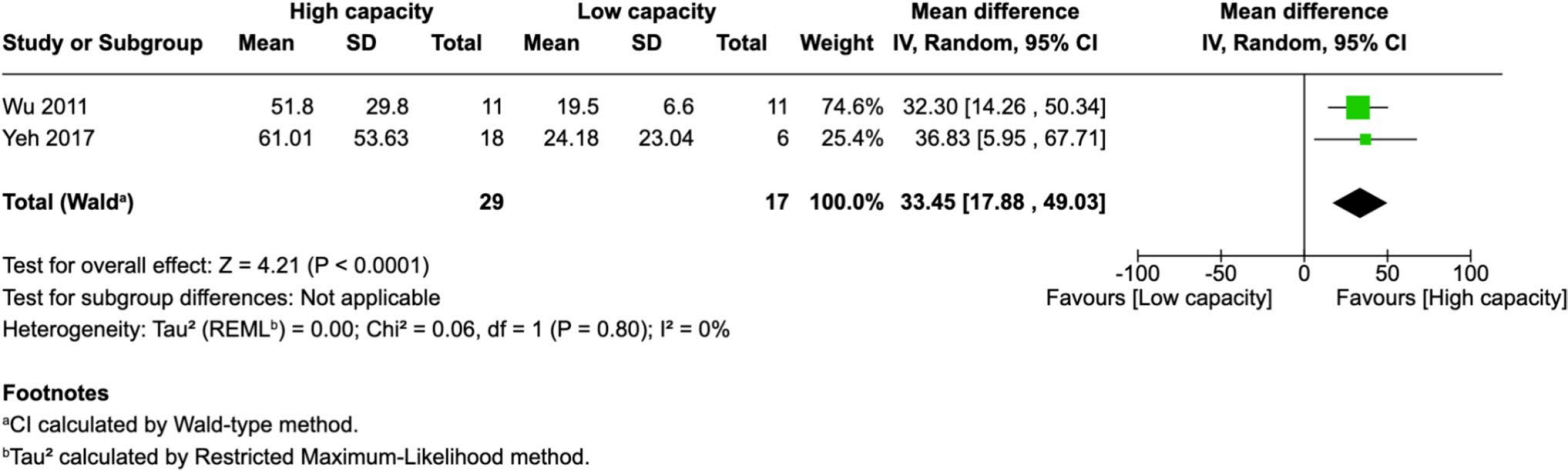
Forest plot showing that higher STA EDV is a significant predictor of high bypass capacity within a timeframe of ≤ 2 weeks

**Fig. 4:**
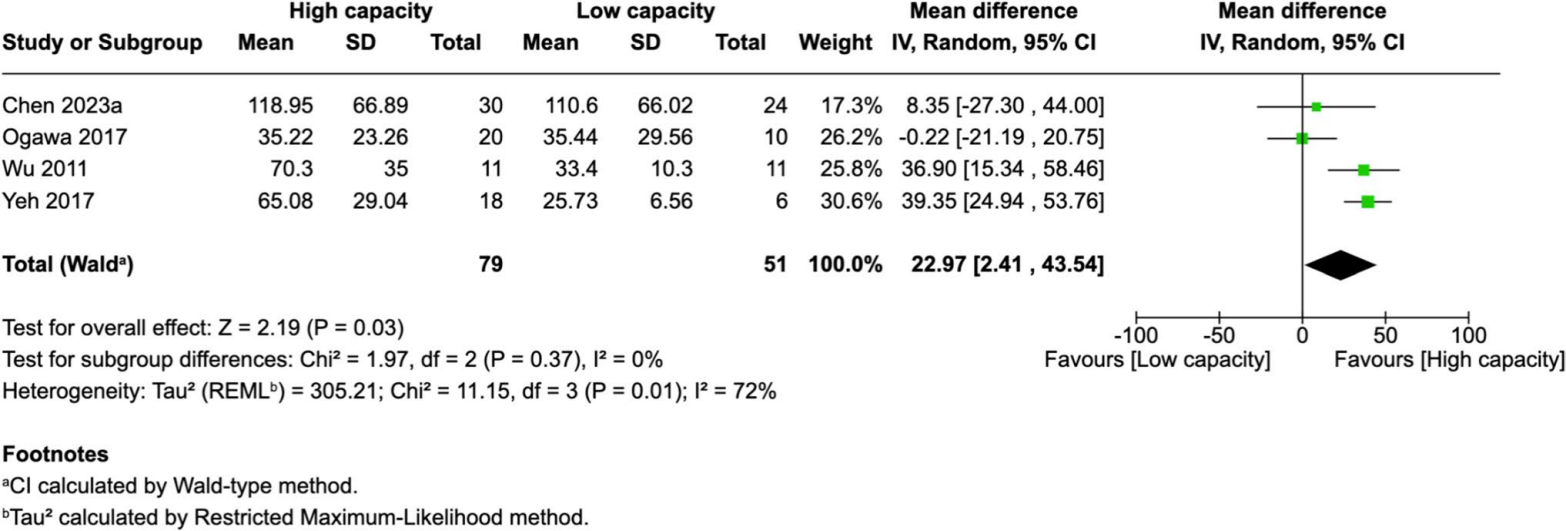
Forest plot showing that higher STA MFV is a significant predictor of high bypass capacity within a timeframe of ≤ 2 weeks

**Fig. 5:**
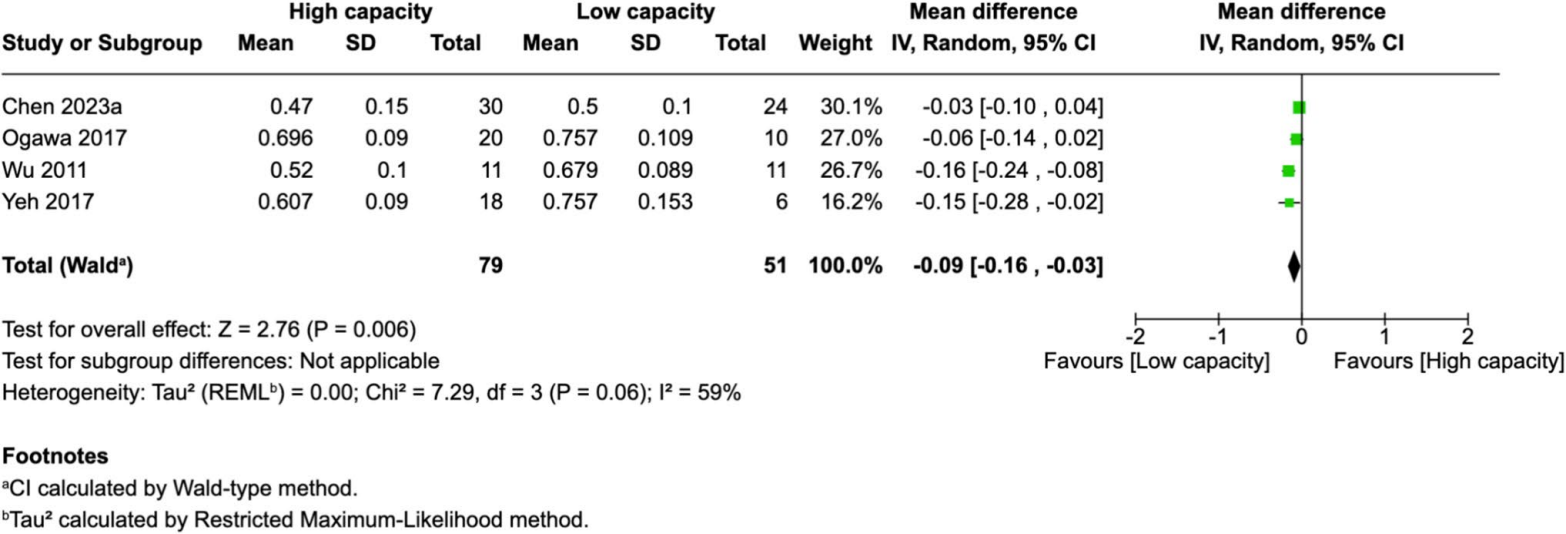
Forest plot showing that lower STA RI is a significant predictor of high bypass capacity within a timeframe of ≤ 2 weeks

**Fig. 6:**
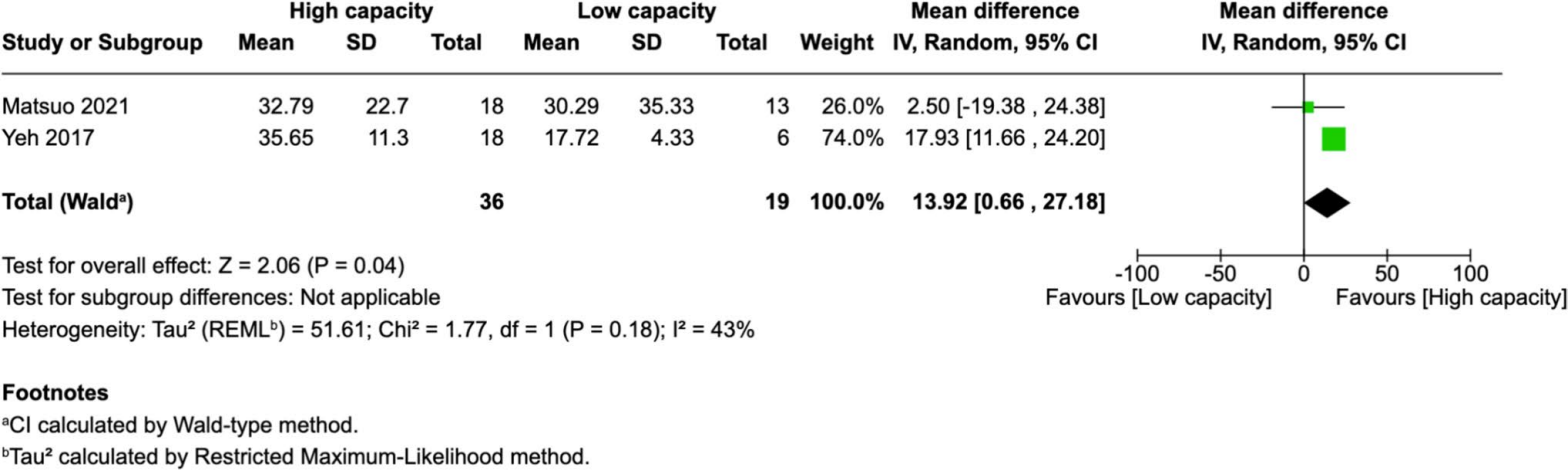
Forest plot showing that higher ECA EDV is a significant predictor of high bypass capacity within a timeframe of ≤ 2 weeks

**Fig. 7:**
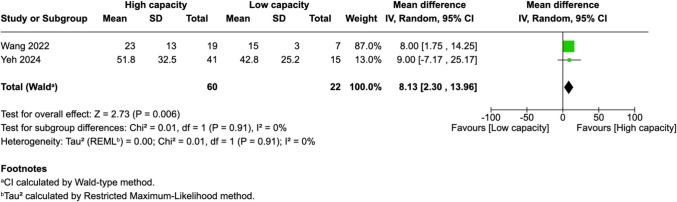
Forest plot showing that higher STA EDV is a significant predictor of high bypass capacity within a timeframe of 3 to 6 months

**Fig. 8:**
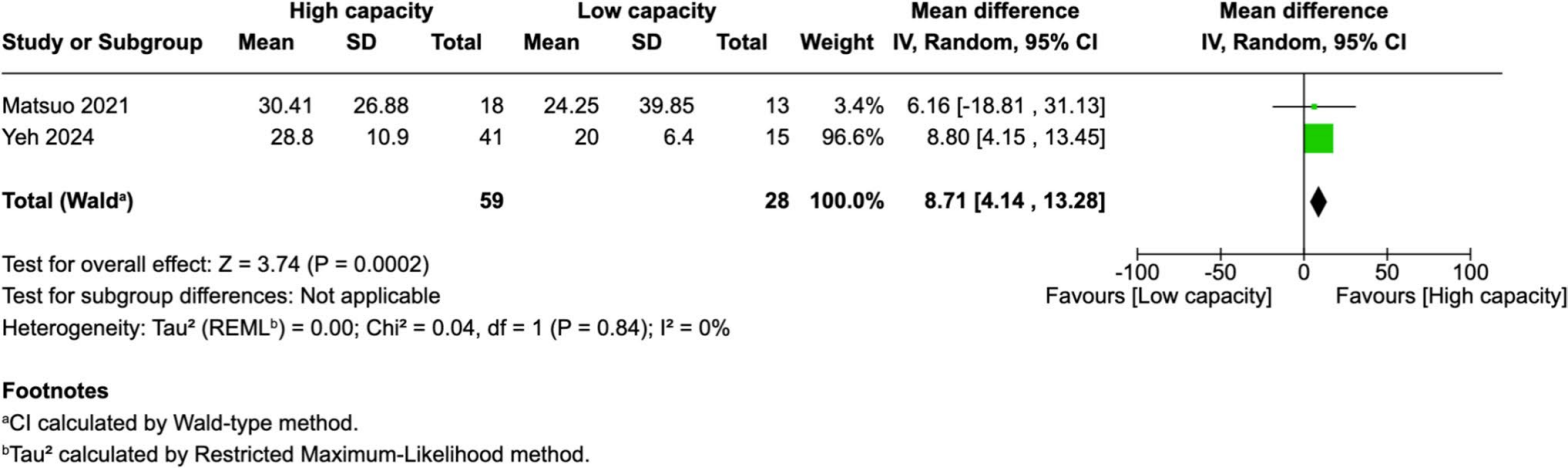
Forest plot showing that higher ECA EDV is a significant predictor of high bypass capacity within a timeframe of 3 to 6 months

**Fig. 9:**
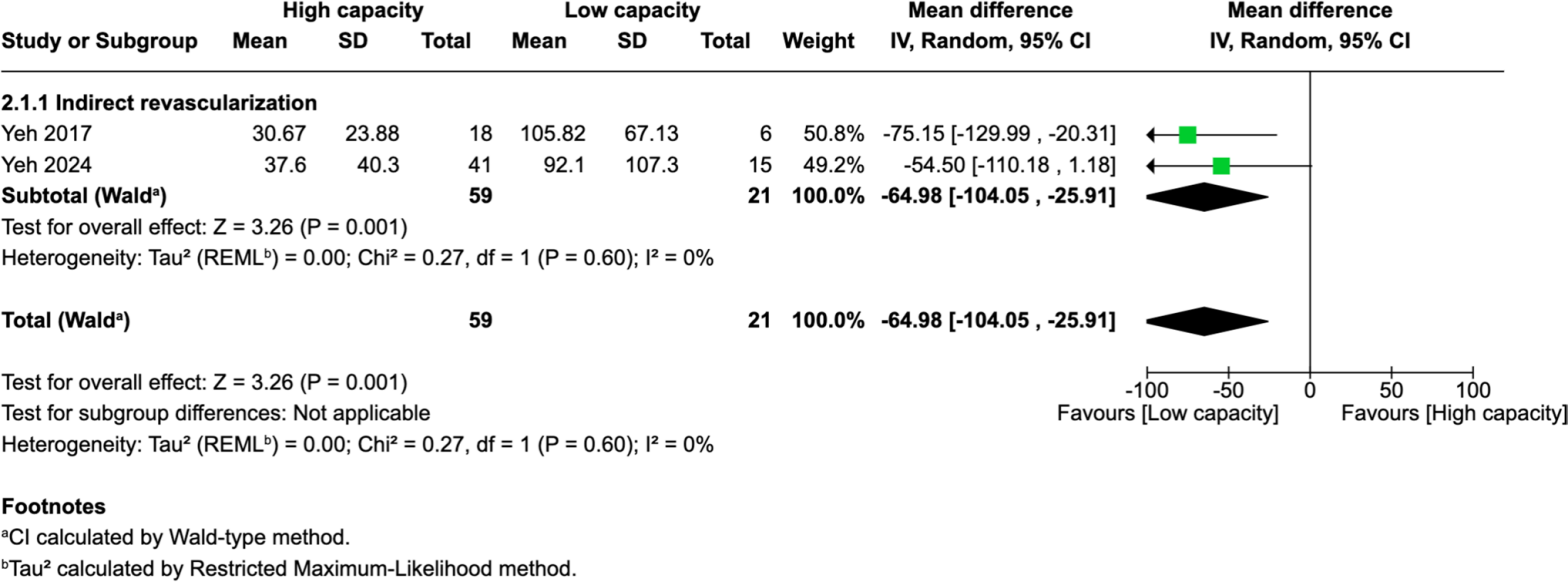
Forest plot showing that lower ACA MFV is a significant predictor of high bypass capacity following indirect surgery within a timeframe of 0 to 3 months

Of 25 assessed variables, five parameters demonstrated significant predictive value within the first 2 weeks, one variable was predictive between 0 and 3 months, and two variables showed predictive value between 3 and 6 months postoperatively. Within 2 weeks of revascularization surgery, higher PSV values (MD = 28.26, 95% CI [14.79–41.74], p < 0.0001) and higher EDV values (MD = 33.45, 95% CI [17.88–49.03], p < 0.0001) in the STA were significantly associated with high-capacity bypass (Fig. 2 and 3). Similarly, increased MFV measured in the STA was predictive of high bypass capacity (MD = 22.97, 95% CI [2.41–43.54], *p* = 0.03, Fig. 4). The RI was significantly lower in high capacity bypasses (MD = −0.09, 95% CI [−0.16 – −0.03], *p* = 0.006, Fig. 5). Additionally, ECA EDV was significantly elevated in the high capacity group during this early period (MD = 13.92, 95% CI [0.66–27.18], *p* = 0.04, Fig. 6). At 3–6 months, STA PSV remained elevated, predicting high bypass capacity (MD = 8.13, 95% CI [2.30—13.96], *p* = 0.006, Fig. 7). Increased EDV in the ECA was another parameter at 3–6 months predicting the high capacity bypass (MD = 8.71, 95% CI [4.14—13.28], *p* = 0.0002, Fig. 8). At a 0–3 months period, a decrease in ACA MFV was observed after indirect surgery (MD = −64.98, 95% CI [−104.05 – −25.91], *p* = 0.001) in high capacity bypasses (Fig. 9).

### Variables lacking association with high bypass capacity

Of the 25 examined variables, the following were not statistically significant: STA PI, ICA PSV, ICA PI, ICA MFV, ICA EDV, ECA PSV, ECA PI, ECA MFV within the first 2 weeks, ACA PI, MCA MFV, MCA PI, PCA MFV, PCA PI at 0–3 months, and STA RI, STA MFV, STA diameter, and ECA MFV at 3–6 months (Supplementary Fig. 2–18). Table 3. summarizes the results of statistical analysis for all assessed variables.

**Table 3:** Summary of statistical analysis for all assessed parameters comparing high and low bypass capacity at different time frames postoperatively

Artery	Parameter	MD	95% CI	*p*-value
*2 weeks period*				
STA	PSV (cm/s)	28.26	14.79; 41.74	< 0.0001*
	EDV (cm/s)	33.45	17.88; 49.03	< 0.0001*
	RI	−0.09	−0.16; −0.03	0.006*
	PI	−0.27	−0.64; 0.10	0.15
	MFV (ml/min)	22.97	2.41; 43.54	0.03*
ECA	PSV (cm/s)	8.14	−19.67; 35.95	0.57
	EDV (cm/s)	13.92	0.66; 27.18	0.04*
	PI	0.84	−0.83; 2.50	0.33
	MFV (ml/min)	2.56	−38.71; 43.83	0.90
ICA	PSV (cm/s)	−3.12	−23.84; 17.61	0.77
	EDV (cm/s)	−3.77	−12.74; 5.19	0.41
	PI	0.20	−0.32; 0.71	0.45
	MFV (ml/min)	−6.01	−24.02; 12.01	0.51
*3–6 months*				
STA	EDV (cm/s)	8.13	2.30; 13.96	0.006*
	RI	−0.04	−0.08; −0.00	0.05
	MFV (ml/min)	4.48	−20.22; 29.17	0.72
	diameter (mm)	−0.08	−0.45; 0.30	0.69
ECA	EDV (cm/s)	8.71	4.14; 13.28	0.0002*
	MFV (ml/min)	31.49	−24.72; 87.69	0.27
*0–3 months*				
ACA	MFV (ml/min)	−64.98	−104.05; −25.91	0.001*
	PI	0.02	−0.08; 0.11	0.74
MCA	MFV (ml/min)	−35.30	−119.55; 48.94	0.41
	PI	0.02	−0.09; 0.13	0.70
PCA	MFV (ml/min)	11.87	−23.72; 47.46	0.51
	PI	0.03	−0.11; 0.18	0.66

* statistically significant. *ACA* anterior cerebral artery, *ECA* external carotid artery, *EDV* end-diastolic velocity, *ICA* internal carotid artery, *MCA* middle cerebral artery, *MFV* mean flow velocity, *PCA* posterior cerebral artery, *PSV* peak systolic velocity, *STA* superficial temporal artery

## Discussion

The goal of revascularization surgery in MMD is to prevent recurrent ischemic stroke by improving CBF to hypoperfused regions or redirecting it around stenosed arteries [4, 19]. However, a number of patients still develop inadequate collateral circulation or compromised bypass patency, leading to ischemic complications. Yeh et al. reported that up to 27% of patients experienced PIEs within the first year post-surgery, reflecting compromised bypass function [24]. With its unparalleled spatial and temporal resolution, DSA remains the gold standard in evaluating EC-IC bypass patency and neovascularization following surgery in MMD. However, its invasive nature, radiation exposure, risk of contrast-induced nephropathy, and frequent need for anesthesia limit its routine use, particularly in pediatric and critically ill patients [26]. Early identification of suboptimal bypass outcomes requires a noninvasive, cost-effective tool that enables timely decision-making.

Previous studies have reported serial US assessments in direct, indirect, and combined bypass procedures [13, 21, 26]. Our findings support the US as a tool that reliably capture hemodynamic changes in extracranial vessels predictive of bypass capacity, with applicability across surgical approaches. Studies suggest that direct revascularization leads to earlier detectable hemodynamic improvements, while indirect procedures—dependent on gradual neovascularization—may show meaningful changes after approximately three months, when collateral formation reaches a plateau [15, 24]. Our study addresses this timeline, highlighting the importance of repeated evaluations during follow-up.

To our knowledge, this is the first meta-analysis to comprehensively evaluate ultrasound parameters as predictors of bypass capacity in MMD. Our findings suggest US can accurately identify high capacity bypasses and serve as a frontline tool in postoperative assessment. Integrating US into follow-up protocols could enable earlier detection of suboptimal outcomes, reduce reliance on invasive imaging, and support more personalized, outcome-driven management strategies.

### Ultrasound predictors within 2 weeks

The early postoperative period offers a critical window to assess bypass efficacy and collateral formation. Due to its anatomical proximity, hemodynamic responsiveness, and direct connection to intracranial vessels, the STA offers a reliable target for early postoperative evaluation [12]. Our study found that higher PSV (MD = 28.26, p < 0.0001), EDV (MD = 33.45, p < 0.0001), and MFV (MD = 22.97, *p* = 0.03) were predictive of high bypass capacity within two weeks post-surgery, reflecting increased and sustained CBF through the anastomosis. In previous studies, Kim et al. reported that early postoperative increases of > 47.5% in mean flow rate or > 15% in STA diameter predicted good patency on DSA, while Hirai et al. found that STA diameter and MFV significantly increased after EC-IC bypass, with an MFV > 48.5 cm/s showing high diagnostic accuracy for sufficient CBF [8, 11]. Although STA diameter was not significant in our analysis, likely due to sample size limitations, it remains a relevant structural correlate of blood flow. Evidence suggests that combining anatomical and functional markers enhances predictive accuracy [3, 18]. Physiologically, elevated flow velocities likely reflect sustained cerebral perfusion through a patent anastomosis, facilitating early collateral recruitment [18]. According to principles of fluid dynamics, when the flow rate increases, resistance decreases [22]. Therefore, in successful bypass procedures, the vascular resistance distal to the anastomosis, usually elevated in ischemic brain regions, decreases as revascularization reestablishes adequate blood flow to these areas. In our study, high-capacity bypasses had significantly lower RI (MD = −0.09, *p* = 0.006), consistent with this mechanism. Wu et al. similarly noted that increased STA flow with reduced RI reflect anastomotic patency [22]. Increased PSV and EDV accompanied by decreased RI suggest effective extracranial-to-intracranial flow [3]. Dong et al. provided a high-resolution view of hemodynamic changes within the first 10 days post-surgery, demonstrating that PSV, EDV, and RI all changed significantly compared to preoperative levels, with EDV peaking around day 5 and RI reaching its lowest value by day 6. PSV fluctuated over time, while daily changes in EDV and RI were significant throughout the first week. The drop in RI was primarily linked to increase in EDV, further supporting the interpretation that rising diastolic flow reflects effective perfusion [5]. These hemodynamic fluctuations highlight the dynamic adaptation of cerebral circulation and the value of US to daily monitoring of bypass function. Ogawa et al. observed that PSV, EDV, and MFV continued to rise gradually for up to 6–12 months in good responders after indirect bypass, aligning with the delayed timeline of neovascularization [15]. Our findings demonstrate that important prognostic information can already be captured within the first two weeks, supporting the value of early ultrasound assessment following indirect and direct surgery. The magnitude of hemodynamic changes is more pronounced in the STA than in other extracranial arteries, such as the ECA. Notably, our study identifies elevated ECA EDV within the first two weeks postoperatively as a predictor of high bypass capacity with the MD = 13.92 (*p* = 0.04), suggesting that early flow augmentation in the ECA may indicate effective collateral engagement. Such values align with the results of Kraemer et al. who reported a 143% increase in STA EDV compared to a 50% increase in the ECA within 30 days of surgery, emphasizing the STA’s sensitivity to cerebral revascularization effects [12]. However, changes in US parameters in both STA and ECA were well associated with angiographic Matsushima grades, providing reliable insights into collateral status [6, 26].

### Ultrasound predictors in the 3–6 months

The 3–6 months postoperative period is associated with reaching the plateau with collateral development and bypass hemodynamics stabilization, making it important for monitoring bypass maturation and collateral integration [21]. In our study, both STA EDV (MD = 8.13, *p* = 0.006) and ECA EDV (MD = 8.71, *p* = 0.0002) remained significantly elevated in high-capacity bypasses during this period. Sustained elevation of EDV likely reflects well developed collateral circulation, particularly following indirect bypass procedures such as EDAS, where long-term neovascularization reduces reliance on the graft itself. Increased EDV in the ECA suggests continued collateral supply through ECA territories, indicating successful angiogenesis stimulated by surgical intervention [9]. Elevated STA EDV corresponds to a low-resistance vascular bed and enhanced blood flow through the graft, signifying its functional integration into cerebral circulation [3]. Supporting these findings, Yeh et al. showed that hemispheres with PIEs at 3 months had lower EDV and FV in the STA and occipital artery (OA), as well as higher RI in the ECA, highlighting the prognostic value of extracranial hemodynamic parameters [24]. In another study, the same group observed significant increases in PSV, EDV, and MFV in the ipsilateral ECA and STA at 6 months, alongside reduced RI [23]. Although our dataset did not allow for analysis of PSV or RI at this time period, previous research affirms their clinical utility [23, 24]. Wang et al. further identified an STA EDV > 16.5 cm/s as a highly sensitive and specific indicator of sufficient collateral formation between 3–6 months postoperatively [21]. Ogawa et al. highlighted that the absence of increases in PSV, EDV, or MFV at 3 months postoperatively predicted poor response to indirect bypass [15]. Similarly, increased ECA EDV at 8 months was associated with favorable angiographic outcomes, reinforcing the long-term prognostic value of extracranial US parameters [26]. Notably, EDV and PSV values may decline in later follow-up compared to early postoperative readings, which likely reflects hemodynamic stabilization and diminished reliance on the bypass as intracranial collaterals mature [22]. These patterns further highlight the need for studying hemodynamics of bypass through standardized follow-up protocols. Although some evidence identifies peripheral ECA resistance as a predictor of collateral status, we were unable to assess its significance due to limited data availability [13]. Future research should incorporate broader hemodynamic profiling across extracranial arteries like the ECA and ICA and seek validated thresholds to guide clinical decisions. Longitudinal studies correlating ultrasound findings with angiographic and clinical outcomes will be essential for refining standardized postoperative monitoring strategies.

### Ultrasound parameters of intracranial arteries at 0–3 months

The hemodynamic profiles of extracranial and intracranial arteries differ fundamentally due to their vascular resistance characteristics. Extracranial vessels such as the STA and ECA belong to a high-resistance system characterized by low diastolic flow velocity and high PI. In contrast, intracranial arteries, such as the ACA, are components of a low-resistance system, typically exhibiting higher diastolic flow and lower PI reflecting their role in maintaining constant cerebral perfusion [12]. Yeh et al. found that both the change in PSV and MFV in the ACA progressively increased from Matsushima grade A (good collateral formation) to grade C (poor collateral formation). Grade C patients exhibited the highest elevations in ACA velocities, suggesting a compensatory hyperdynamic response due to insufficient revascularization in the MCA territory [26]. The ACA often serves as a major source of collateral flow when the MCA territory is ischemic. In the setting of indirect revascularization, the ACA frequently compensates for insufficient MCA perfusion, as reflected in elevated ACA MFV in patients with poor collateralization [20, 26]. Other studies have similarly reported postoperative ACA MFV elevations in patients with suboptimal collateral development [7, 24]. Conversely, patients with Matsushima grades A and B exhibited lower postoperative ACA and MCA velocities compared to baseline, suggesting effective bypass without stenosis [26]. In our analysis, a significant decrease in ACA MFV was observed during the 0–3 month period following indirect bypass in high-capacity cases (MD = –64.98, *p* = 0.001), which likely reflects redistribution of blood flow following effective revascularization. In patients with favorable Matsushima grades, newly established collaterals offload flow demand from the ACA, reducing its hemodynamic burden [24, 26]. As shown by Ding et al., ACA MFV remained elevated or showed delayed normalization post-bypass in patients with impaired collateral formation, with implications for predicting postoperative infarction [5]. Yeh et al. also found that although no significant differences in intracranial parameters were observed between hemispheres with and without PIEs, there was a trend toward higher postoperative ACA MFV in PIE-positive hemispheres, suggesting that persistent ACA hyperperfusion reflects bypass insufficiency [24]. Monitoring ACA MFV via TCD or duplex ultrasound during the early postoperative period provides valuable insight into the success of revascularization, serving as an indirect marker of bypass efficacy and collateral development.

## Limitations

This study has several limitations. First, the small sample sizes may potentially increase the risk of bias and sampling error. This limitation could have also obscured the significance of certain ultrasound parameters that have shown associations with surgical outcomes in prior literature but were not significant in our analysis, such as the PI, which reportedly decreases alongside RI in successful bypasses [26]. Due to the limited number of eligible studies, we were unable to perform subgroup analyses and assess heterogeneity between surgical approaches. Although direct and indirect revascularization share the common goal of ensuring CBF and are frequently combined in clinical practice, they differ in technique and underlying mechanism [26]. While our study comprehensively evaluates US markers of both bypass patency and collateral development across all surgical strategies, it remains essential to acknowledge these procedural differences and to study their effects in future research. Age-stratified analysis was not feasible due to limited data, while evidence suggests that ultrasonographic responses may differ between adults and pediatric patients. Yeh et al. reported that children exhibited greater postoperative changes in MFV and volume in the ECA and STA following indirect procedures, whereas certain STA parameters were more predictive of ischemic events in adults [25]. These age-related hemodynamic differences require further investigation in future studies. A key source of heterogeneity comes from the lack of standardized criteria for assessing bypass capacity. In direct bypass, patency usually refers to an anastomotic function, while in indirect approach, success is defined by collateral development, often assessed using the Matsushima grading scale [14]. However, these criteria are often prone to subjective interpretation. Definitions varied across studies, with some using the absence of PIEs or non-specific assessments of flow adequacy. Though we applied a random-effects model to address variability, this inconsistency may introduce interpretive limitations. Limited data also constrained analysis of certain arteries and parameters. For some arteries, only a few US metrics were reported. Intracranial arteries assessment (ACA, MCA, PCA) was limited to studies on indirect bypass, precluding comparison with direct approaches and limiting insight into broader cerebral hemodynamics after surgery. Lack of data on posterior circulation did not allow for comparison of US findings with modalities such as MRA, which demonstrated decreased arterial flow in the posterior circulation after bypass surgery, interpreted as a shift in hemodynamic burden from stenosed or occluded vessels [10]. Future studies should include broader arterial profiling and compare US findings with MRA and CTA to better represent global flow changes and bypass efficacy. The timing of postoperative ultrasound varied considerably, complicating efforts to establish a consistent timeline of hemodynamic changes. Moreover, few studies extended follow-up beyond 6 months, identifying a critical need for long-term studies employing standardized protocols for both the timing and frequency of US and DSA to support more accurate prognostication, and help define optimal postoperative monitoring strategies. Ultrasound’s operator-dependent nature introduces further variability, influenced by examiner experience, probe placement, and equipment quality. The studies differed in protocols, target arteries, measured parameters, and follow-up schedules, further complicating analysis. Additionally, we could not explore associations between US parameters and cerebral perfusion metrics such as SPECT or CT perfusion, limiting the physiological context of our findings. Lastly, all the studies included the Asian population, where MMD is most prevalent, but may limit the generalizability of our findings. However, available data from European cohorts suggest comparable early hemodynamic changes observed in European cohorts undergoing EC-IC bypass for MMD and other occlusive diseases [12]. Future studies should validate the clinical utility of US in larger and more diverse populations, using standardized protocols with long-term clinical and radiographic follow-up.

## Conclusions

This study demonstrates the utility of US as a noninvasive and cost-effective tool for evaluating bypass function and collateral development following revascularization in MMD. Elevated STA PSV, EDV, and MFV within two weeks postoperatively, along with lower RI values, and increased ECA EDV were predictive of high bypass capacity. At 3–6 months, sustained increases in STA PSV and ECA EDV were also associated with favorable outcomes, while decreased ACA MFV at 0–3 months reflected successful adaptation following indirect bypass. Our results indicate that US enables accurate detection of high-capacity bypasses and may serve as a first-line modality in the postoperative assessment of MMD patients. Ultrasound may help quickly identify patients at higher risk of bypass insufficiency who should undergo confirmatory DSA, thereby minimizing unnecessary invasive procedures. Routine incorporation of US into follow-up protocols could improve early risk stratification and guide individualized management. Future research should define diagnostic thresholds, standardize the assessment protocols and follow-up timing, and validate US parameters against angiographic and clinical outcomes to strengthen postoperative care in MMD.

## Supplementary Information

Below is the link to the electronic supplementary material.

ESM1 (PDF 2.13 MB)

## Data Availability

No datasets were generated or analysed during the current study.
