# Supplementary material for: Ultrasonographic assessment of bypass capacity after revascularization surgery in moyamoya disease: a systematic review and single-arm meta-analysis
Source: Acta Neurochir (Wien). 2025 Sep 10;167(1):242. doi: 10.1007/s00701-025-06658-6 (PMC12423130; doi:10.1007/s00701-025-06658-6)
Supplement: Supplementary file 1 — (PDF 2.13 MB) [file 701_2025_6658_MOESM1_ESM.pdf]

Acta Neurochirurgica

Supplementary material

Ultrasonographic assessment of bypass capacity after revascularization surgery in moyamoya disease: a systematic review and single-arm meta-analysis

Natalia Anna Koc[1], Maurycy Rakowski, MD[1], Samuel D. Pettersson[1,2],
Paulina Skrzypkowska, MD[1], Tomasz Szmuda, MD, PhD[1], Piotr Zieliński, MD, PhD[1]

1 - Department of Neurosurgery, Medical University of Gdańsk, Gdańsk, Poland
2 - Neurosurgical Service, Beth Israel Deaconess Medical Center, Harvard Medical School, Boston, MA, USA

Corresponding author:

Piotr Zieliński, MD, PhD, *Department of Neurosurgery, Medical University of Gdańsk, Gdańsk, Poland*

Corresponding author's email:

pziel@gumed.edu.pl

Supplementary Table 1 The included studies and their quality rating using the modified Newcastle-Ottawa Scale. DSA – digital subtraction angiography.

	Selection				Comparability		Outcome				
Study	Representativeness of the exposed cohort	Selection of the non exposed cohort	Ascertainment of exposure	Demonstration that outcome of interest was not present at start of study	Study controls (High capacity / Low capacity)	Study controls for any additional factor (ultrasound / DSA)	Assessment of outcome	Was follow-up time long enough for outcomes to occur (min. 14 days)	Adequacy of follow up of cohorts (loss-to-follow-up 20%)	Total score	Rating
Yeh 2017	1	0	1	1	1	1	1	1	1	8/9	high quality
Wang 2022	1	0	1	1	1	1	1	1	1	8/9	high quality
Ogawa 2017	1	0	1	1	1	0	1	1	1	7/9	high quality
Matsuo 2021	1	0	1	1	1	0	1	1	1	7/9	high quality
Chen 2023a	1	0	1	1	1	0	1	1	1	7/9	high quality
Yeh 2024	1	0	1	1	1	1	1	1	1	8/9	high quality
Wu 2011	1	0	1	1	1	0	1	1	1	7/9	high quality
Chen 2023b	1	0	1	1	1	0	1	1	1	7/9	high quality

Supplementary Fig. 1 Funnel plot indicating publication bias of being present. SE – standard error; OR – odds ratio.

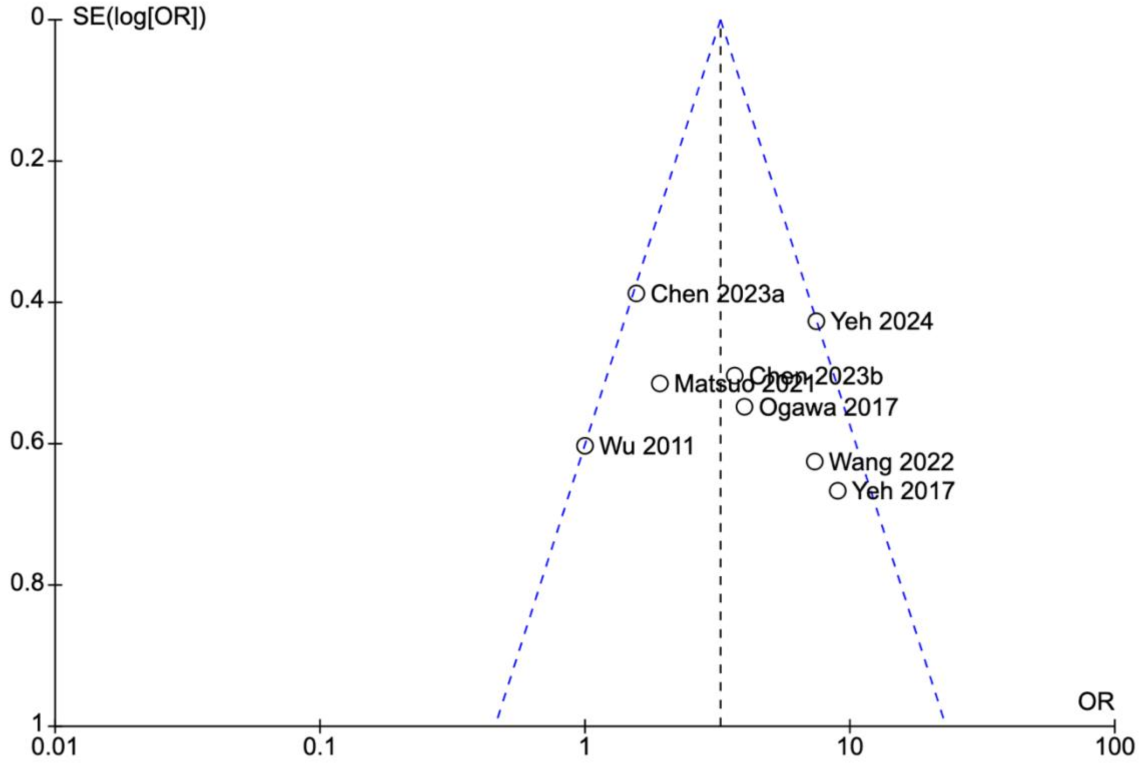

Forest plots for a timeframe of ≤ 2 weeks:

Supplementary Fig. 2 Forest plot showing that STA PI is a non-significant predictor of high bypass capacity within a timeframe of ≤ 2 weeks. STA – superficial temporal artery; PI – pulsatility index; IV – inverse variance; SD – standard deviation; CI – confidence interval.

Study or Subgroup	High capacity Mean	SD	Total	Low capacity Mean	SD	Total	Weight	Mean difference IV, Random, 95% CI
Chen 2023a	0.87	1.25	30	0.7	0.25	24	25.7%	0.17 [-0.29 , 0.63]
Chen 2023b	1.07	0.27	23	1.25	0.39	12	35.6%	-0.18 [-0.43 , 0.07]
Wu 2011	0.79	0.35	11	1.28	0.21	11	35.8%	-0.49 [-0.73 , -0.25]
Yeh 2017	2	0.71	18	4.49	2.56	6	3.0%	-2.49 [-4.56 , -0.42]
**Total (Wald[a])**			**82**			**53**	**100.0%**	**-0.27 [-0.64 , 0.10]**

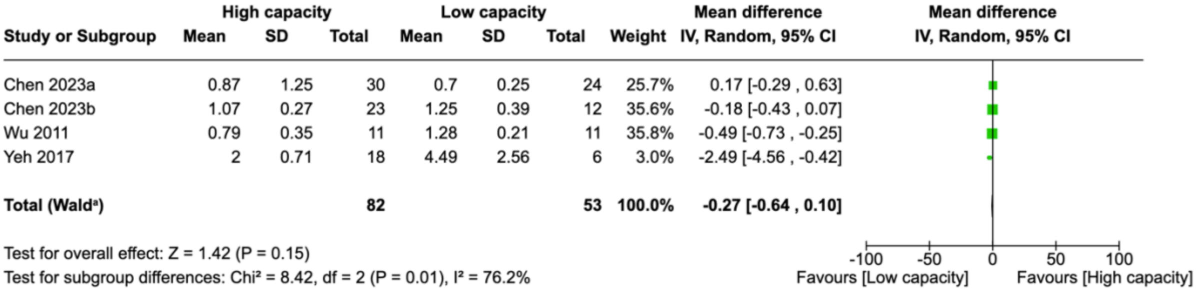

Test for overall effect: Z = 1.42 (P = 0.15)
Test for subgroup differences: Chi^2 = 8.42, df = 2 (P = 0.01), I^2 = 76.2%
Heterogeneity: Tau^2 (REML[b]) = 0.09; Chi^2 = 11.59, df = 3 (P = 0.009); I^2 = 72%

Footnotes
[a]CI calculated by Wald-type method.
[b]Tau^2 calculated by Restricted Maximum-Likelihood method.

Supplementary Fig. 3 Forest plot showing that ICA PSV is a non-significant predictor of high bypass capacity within a timeframe of ≤ 2 weeks. ICA – internal carotid artery; PSV – peak systolic velocity; IV – inverse variance; SD – standard deviation; CI – confidence interval.

Study or Subgroup	High capacity Mean	SD	Total	Low capacity Mean	SD	Total	Weight	Mean difference IV, Random, 95% CI
Matsuo 2021	52.4	64.13	18	63.71	58.77	13	22.6%	-11.31 [-54.88 , 32.26]
Yeh 2017	75.09	20.48	18	75.81	26.97	6	77.4%	-0.72 [-24.28 , 22.84]
**Total (Wald[a])**			**36**			**19**	**100.0%**	**-3.12 [-23.84 , 17.61]**

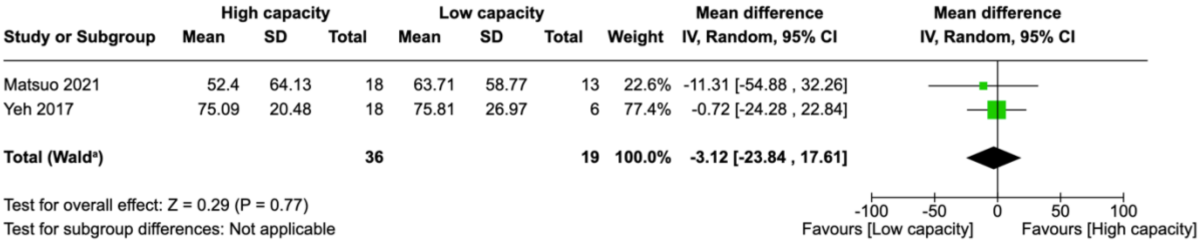

Test for overall effect: Z = 0.29 (P = 0.77)
Test for subgroup differences: Not applicable
Heterogeneity: Tau^2 (REML[b]) = 0.00; Chi^2 = 0.18, df = 1 (P = 0.68); I^2 = 0%

Footnotes
[a]CI calculated by Wald-type method.
[b]Tau^2 calculated by Restricted Maximum-Likelihood method.

Supplementary Fig. 4 Forest plot showing that ICA PI is a non-significant predictor of high bypass capacity within a timeframe of ≤ 2 weeks. ICA – internal carotid artery; PI – pulsatility index; IV – inverse variance; SD – standard deviation; CI – confidence interval.

Study or Subgroup	High capacity Mean	SD	Total	Low capacity Mean	SD	Total	Weight	Mean difference IV, Random, 95% CI
Matsuo 2021	1.41	2.23	18	1.26	1.16	13	18.2%	0.15 [-1.06 , 1.36]
Yeh 2017	1.9	0.61	18	1.69	0.62	6	81.8%	0.21 [-0.36 , 0.78]
**Total (Wald[a])**			**36**			**19**	**100.0%**	**0.20 [-0.32 , 0.71]**

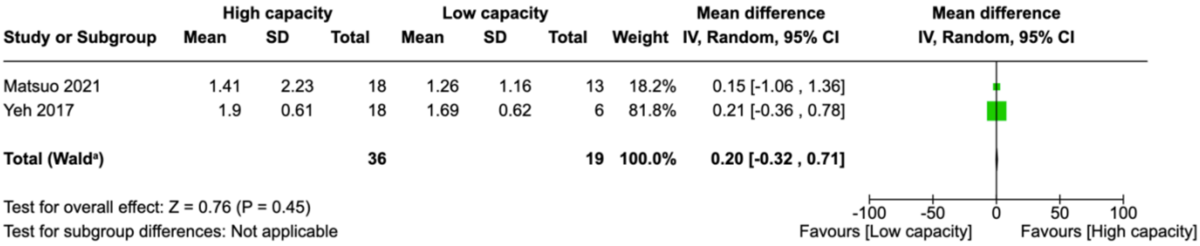

Test for overall effect: Z = 0.76 (P = 0.45)
Test for subgroup differences: Not applicable
Heterogeneity: Tau^2 (REML[b]) = 0.00; Chi^2 = 0.01, df = 1 (P = 0.93); I^2 = 0%

Footnotes
[a]CI calculated by Wald-type method.
[b]Tau^2 calculated by Restricted Maximum-Likelihood method.

Supplementary Fig. 5 Forest plot showing that ICA MFV is a non-significant predictor of high bypass capacity within a timeframe of ≤ 2 weeks. ICA – internal carotid artery; MFV – mean flow velocity; IV – inverse variance; SD – standard deviation; CI – confidence interval.

Study or Subgroup	High capacity Mean	SD	Total	Low capacity Mean	SD	Total	Weight	Mean difference IV, Random, 95% CI
Matsuo 2021	29.19	32.15	18	33.86	20.14	13	95.4%	-4.67 [-23.12 , 13.78]
Yeh 2017	148.17	59.68	18	182.15	99.52	6	4.6%	-33.98 [-118.25 , 50.29]
**Total (Wald[a])**			**36**			**19**	**100.0%**	**-6.01 [-24.04 , 12.01]**

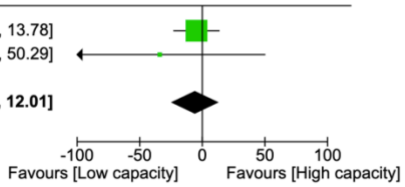

Test for overall effect: Z = 0.65 (P = 0.51)
Test for subgroup differences: Chi² = 0.44, df = 1 (P = 0.51), I² = 0%
Heterogeneity: Tau² (REML[b]) = 0.00; Chi² = 0.44, df = 1 (P = 0.51); I² = 0%

Footnotes
[a]CI calculated by Wald-type method.
[b]Tau² calculated by Restricted Maximum-Likelihood method.

Supplementary Fig. 6 Forest plot showing that ICA EDV is a non-significant predictor of high bypass capacity within a timeframe of ≤ 2 weeks. ICA – internal carotid artery; EDV – end diastolic velocity; IV – inverse variance; SD – standard deviation; CI – confidence interval.

Study or Subgroup	High capacity Mean	SD	Total	Low capacity Mean	SD	Total	Weight	Mean difference IV, Random, 95% CI
Matsuo 2021	24.56	28.19	18	27.84	29.35	13	18.9%	-3.28 [-23.87 , 17.31]
Yeh 2017	28.15	8.89	18	32.04	11.33	6	81.1%	-3.89 [-13.84 , 6.06]
**Total (Wald[a])**			**36**			**19**	**100.0%**	**-3.77 [-12.74 , 5.19]**

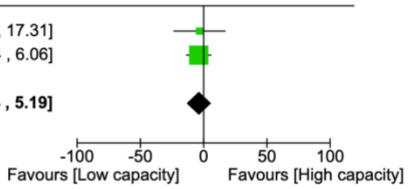

Test for overall effect: Z = 0.83 (P = 0.41)
Test for subgroup differences: Not applicable
Heterogeneity: Tau² (REML[b]) = 0.00; Chi² = 0.00, df = 1 (P = 0.96); I² = 0%

Footnotes
[a]CI calculated by Wald-type method.
[b]Tau² calculated by Restricted Maximum-Likelihood method.

Supplementary Fig. 7 Forest plot showing that ECA PSV is a non-significant predictor of high bypass capacity within a timeframe of ≤ 2 weeks. ECA – external carotid artery; PSV – peak systolic velocity; IV – inverse variance; SD – standard deviation; CI – confidence interval.

Study or Subgroup	High capacity Mean	SD	Total	Low capacity Mean	SD	Total	Weight	Mean difference IV, Random, 95% CI
Matsuo 2021	127.35	70.46	18	132.02	196.1	13	6.2%	-4.67 [-116.13 , 106.79]
Yeh 2017	123.23	25.95	18	114.24	32.61	6	93.8%	8.99 [-19.73 , 37.71]
**Total (Wald[a])**			**36**			**19**	**100.0%**	**8.14 [-19.67 , 35.95]**

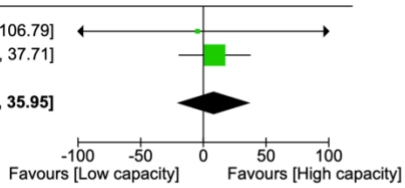

Test for overall effect: Z = 0.57 (P = 0.57)
Test for subgroup differences: Chi² = 0.05, df = 1 (P = 0.82), I² = 0%
Heterogeneity: Tau² (REML[b]) = 0.00; Chi² = 0.05, df = 1 (P = 0.82); I² = 0%

Footnotes
[a]CI calculated by Wald-type method.
[b]Tau² calculated by Restricted Maximum-Likelihood method.

Supplementary Fig. 8 Forest plot showing that ECA PI is a non-significant predictor of high bypass capacity within a timeframe of ≤ 2 weeks. ECA – external carotid artery; PI – pulsatility index; IV – inverse variance; SD – standard deviation; CI – confidence interval.

	High capacity			Low capacity				Mean difference
**Study or Subgroup**	**Mean**	**SD**	**Total**	**Mean**	**SD**	**Total**	**Weight**	**IV, Random, 95% CI**
Matsuo 2021	1.69	0.98	18	1.69	0.7	13	50.8%	0.00 [-0.59 , 0.59]
Yeh 2017	3.39	1.15	18	1.69	0.62	6	49.2%	1.70 [0.97 , 2.43]
**Total (Wald[a])**			**36**			**19**	**100.0%**	**0.84 [-0.83 , 2.50]**

Mean difference IV, Random, 95% CI

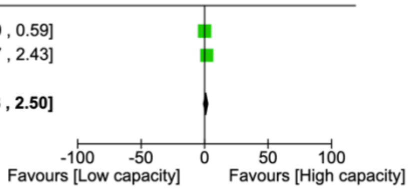

Test for overall effect: Z = 0.98 (P = 0.33)
Test for subgroup differences: Not applicable
Heterogeneity: Tau^2 (REML[b]) = 1.33; Chi^2 = 12.64, df = 1 (P = 0.0004); I^2 = 92%

Footnotes
[a]CI calculated by Wald-type method.
[b]Tau^2 calculated by Restricted Maximum-Likelihood method.

Supplementary Fig. 9 Forest plot showing that ECA MFV is a non-significant predictor of high bypass capacity within a timeframe of ≤ 2 weeks. ECA – external carotid artery; MFV – mean flow velocity; IV – inverse variance; SD – standard deviation; CI – confidence interval.

	High capacity			Low capacity				Mean difference
**Study or Subgroup**	**Mean**	**SD**	**Total**	**Mean**	**SD**	**Total**	**Weight**	**IV, Random, 95% CI**
Matsuo 2021	56.83	33.52	18	57.24	82.77	13	75.2%	-0.41 [-47.99 , 47.17]
Yeh 2017	193.72	50.01	18	182.15	99.52	6	24.8%	11.57 [-71.34 , 94.48]
**Total (Wald[a])**			**36**			**19**	**100.0%**	**2.56 [-38.71 , 43.83]**

Mean difference IV, Random, 95% CI

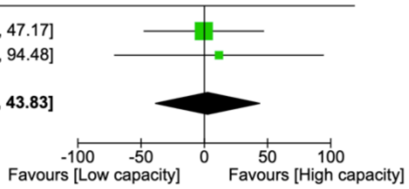

Test for overall effect: Z = 0.12 (P = 0.90)
Test for subgroup differences: Not applicable
Heterogeneity: Tau^2 (REML[b]) = 0.00; Chi^2 = 0.06, df = 1 (P = 0.81); I^2 = 0%

Footnotes
[a]CI calculated by Wald-type method.
[b]Tau^2 calculated by Restricted Maximum-Likelihood method.

Forest plots for a timeframe of 3 to 6 months:

Supplementary Fig. 10 Forest plot showing that STA MFV is a non-significant predictor of high bypass capacity within a timeframe of 3 to 6 months. STA – superficial temporal artery; MFV – mean flow velocity; IV – inverse variance; SD – standard deviation; CI – confidence interval.

	High capacity			Low capacity				Mean difference
**Study or Subgroup**	**Mean**	**SD**	**Total**	**Mean**	**SD**	**Total**	**Weight**	**IV, Random, 95% CI**
Chen 2023a	60.45	45.39	30	95.2	55.51	24	23.0%	-34.75 [-62.26 , -7.24]
Ogawa 2017	44.51	58.99	20	37.75	28.79	10	21.2%	6.76 [-24.65 , 38.17]
Wang 2022	39.47	47.95	19	16.88	8.09	7	25.5%	22.59 [0.21 , 44.97]
Yeh 2024	49.5	24.1	41	32.1	15.1	15	30.4%	17.40 [6.78 , 28.02]
**Total (Wald[a])**			**110**			**56**	**100.0%**	**4.48 [-20.22 , 29.17]**

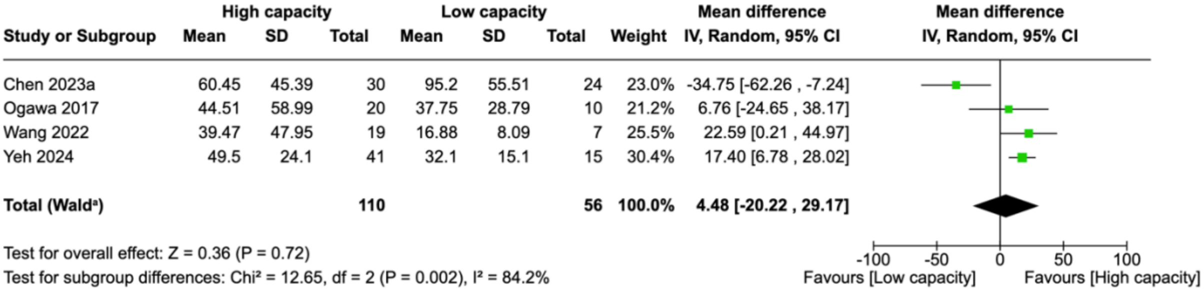

Test for overall effect: Z = 0.36 (P = 0.72)
Test for subgroup differences: Chi^2 = 12.65, df = 2 (P = 0.002), I^2 = 84.2%
Heterogeneity: Tau^2 (REML[b]) = 493.18; Chi^2 = 13.05, df = 3 (P = 0.005); I^2 = 81%

Footnotes
[a]CI calculated by Wald-type method.
[b]Tau^2 calculated by Restricted Maximum-Likelihood method.

Supplementary Fig. 11 Forest plot showing that STA RI is a non-significant predictor of high bypass capacity within a timeframe of 3 to 6 months. STA – superficial temporal artery; RI – resistance index; IV – inverse variance; SD – standard deviation; CI – confidence interval.

	High capacity			Low capacity				Mean difference
**Study or Subgroup**	**Mean**	**SD**	**Total**	**Mean**	**SD**	**Total**	**Weight**	**IV, Random, 95% CI**
Chen 2023a	0.49	0.14	30	0.49	0.12	24	29.3%	0.00 [-0.07 , 0.07]
Ogawa 2017	0.63	0.09	20	0.75	0.15	10	15.3%	-0.12 [-0.22 , -0.02]
Wang 2022	0.62	0.22	19	0.68	0.13	7	8.6%	-0.06 [-0.20 , 0.08]
Yeh 2024	0.64	0.12	41	0.68	0.07	15	46.9%	-0.04 [-0.09 , 0.01]
**Total (Wald[a])**			**110**			**56**	**100.0%**	**-0.04 [-0.08 , -0.00]**

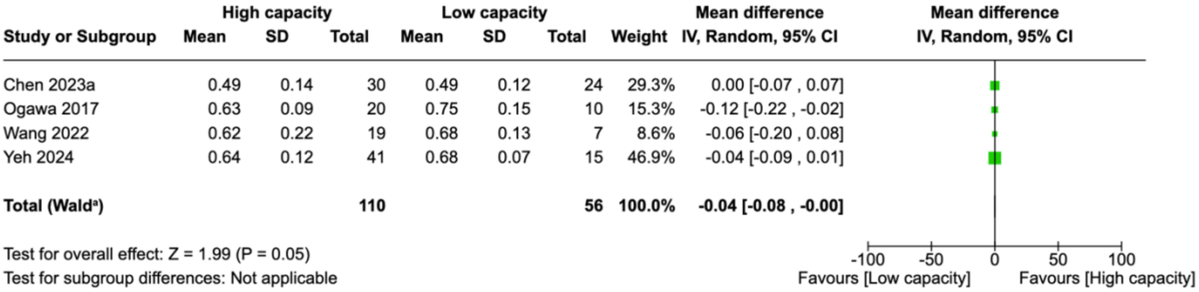

Test for overall effect: Z = 1.99 (P = 0.05)
Test for subgroup differences: Not applicable
Heterogeneity: Tau^2 (REML[b]) = 0.00; Chi^2 = 3.77, df = 3 (P = 0.29); I^2 = 14%

Footnotes
[a]CI calculated by Wald-type method.
[b]Tau^2 calculated by Restricted Maximum-Likelihood method.

Supplementary Fig. 12 Forest plot showing that STA diameter is a non-significant predictor of high bypass capacity within a timeframe of 3 to 6 months. STA – superficial temporal artery; IV – inverse variance; SD – standard deviation; CI – confidence interval.

	High capacity			Low capacity				Mean difference
Study or Subgroup	Mean	SD	Total	Mean	SD	Total	Weight	IV, Random, 95% CI
Chen 2023a	1.66	0.4	30	1.87	0.36	24	67.7%	-0.21 [-0.41 , -0.01]
Wang 2022	2	0.6	19	1.8	0.6	7	32.3%	0.20 [-0.32 , 0.72]
**Total (Wald[a])**			**49**			**31**	**100.0%**	**-0.08 [-0.45 , 0.30]**

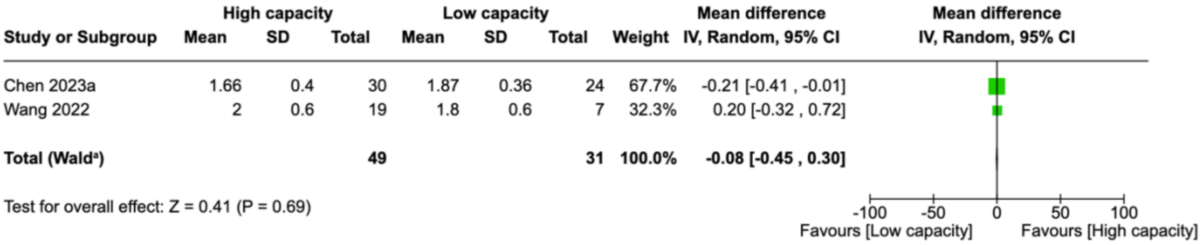

Test for overall effect: Z = 0.41 (P = 0.69)

Heterogeneity: Tau² (REML[b]) = 0.04; Chi² = 2.07, df = 1 (P = 0.15); I² = 52%

Footnotes

[a]CI calculated by Wald-type method.

[b]Tau² calculated by Restricted Maximum-Likelihood method.

Supplementary Fig. 13 Forest plot showing that ECA MFV is a non-significant predictor of high bypass capacity within a timeframe of 3 to 6 months. ECA – external carotid artery; MFV – mean flow velocity; IV – inverse variance; SD – standard deviation; CI – confidence interval.

	High capacity			Low capacity				Mean difference
Study or Subgroup	Mean	SD	Total	Mean	SD	Total	Weight	IV, Random, 95% CI
Matsuo 2021	49.52	44.25	18	49.52	77.4	13	45.3%	0.00 [-46.78 , 46.78]
Yeh 2024	196	83.6	41	138.4	36.7	15	54.7%	57.60 [25.98 , 89.22]
**Total (Wald[a])**			**59**			**28**	**100.0%**	**31.49 [-24.72 , 87.69]**

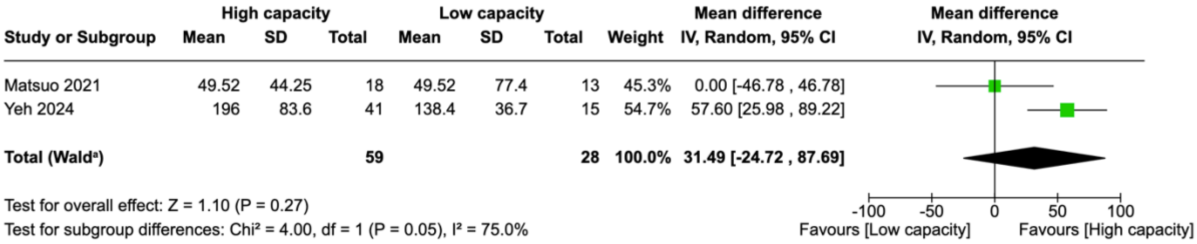

Test for overall effect: Z = 1.10 (P = 0.27)
Test for subgroup differences: Chi² = 4.00, df = 1 (P = 0.05), I² = 75.0%
Heterogeneity: Tau² (REML[b]) = 1243.95; Chi² = 4.00, df = 1 (P = 0.05); I² = 75%

Footnotes

[a]CI calculated by Wald-type method.

[b]Tau² calculated by Restricted Maximum-Likelihood method.

Forest plots for a timeframe of 0 to 3 months:

Supplementary Fig. 14 Forest plot showing that ACA PI is a non-significant predictor of high bypass capacity within a timeframe of 3 to 6 months. ACA – anterior cerebral artery; PI – pulsatility index; IV – inverse variance; SD – standard deviation; CI – confidence interval.

	Experimental			Control				Mean difference
Study or Subgroup	Mean	SD	Total	Mean	SD	Total	Weight	IV, Random, 95% CI
**2.2.1 Indirect revascularization**								
Yeh 2017	0.85	0.29	18	0.82	0.13	6	30.6%	0.03 [-0.14 , 0.20]
Yeh 2024	0.75	0.22	33	0.74	0.16	14	69.4%	0.01 [-0.10 , 0.12]
**Subtotal (Wald[a])**			**51**			**20**	**100.0%**	**0.02 [-0.08 , 0.11]**
Test for overall effect: Z = 0.34 (P = 0.74)								
Heterogeneity: Tau² (REML[b]) = 0.00; Chi² = 0.04, df = 1 (P = 0.85); I² = 0%								
**Total (Wald[a])**			**51**			**20**	**100.0%**	**0.02 [-0.08 , 0.11]**

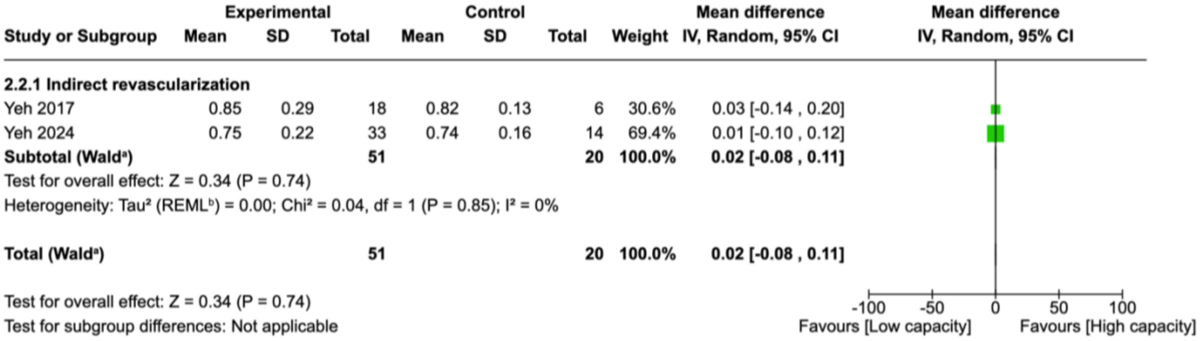

Test for overall effect: Z = 0.34 (P = 0.74)
Test for subgroup differences: Not applicable
Heterogeneity: Tau² (REML[b]) = 0.00; Chi² = 0.04, df = 1 (P = 0.85); I² = 0%

Footnotes
[a]CI calculated by Wald-type method.
[b]Tau² calculated by Restricted Maximum-Likelihood method.

Supplementary Fig. 15 Forest plot showing that MCA MFV is a non-significant predictor of high bypass capacity within a timeframe of 3 to 6 months. MCA – middle cerebral artery; MFV – mean flow velocity; IV – inverse variance; SD – standard deviation; CI – confidence interval.

	High capacity			Low capacity				Mean difference
Study or Subgroup	Mean	SD	Total	Mean	SD	Total	Weight	IV, Random, 95% CI
**2.3.1 Indirect revascularization**								
Yeh 2017	47.26	45.11	18	129.45	89.68	6	45.7%	-82.19 [-156.91 , -7.47]
Yeh 2024	97.5	90.8	41	93.4	95.4	15	54.3%	4.10 [-51.61 , 59.81]
**Subtotal (Wald[a])**			**59**			**21**	**100.0%**	**-35.30 [-119.55 , 48.94]**
Test for overall effect: Z = 0.82 (P = 0.41)								
Heterogeneity: Tau² (REML[b]) = 2592.33; Chi² = 3.29, df = 1 (P = 0.07); I² = 70%								
**Total (Wald[a])**			**59**			**21**	**100.0%**	**-35.30 [-119.55 , 48.94]**

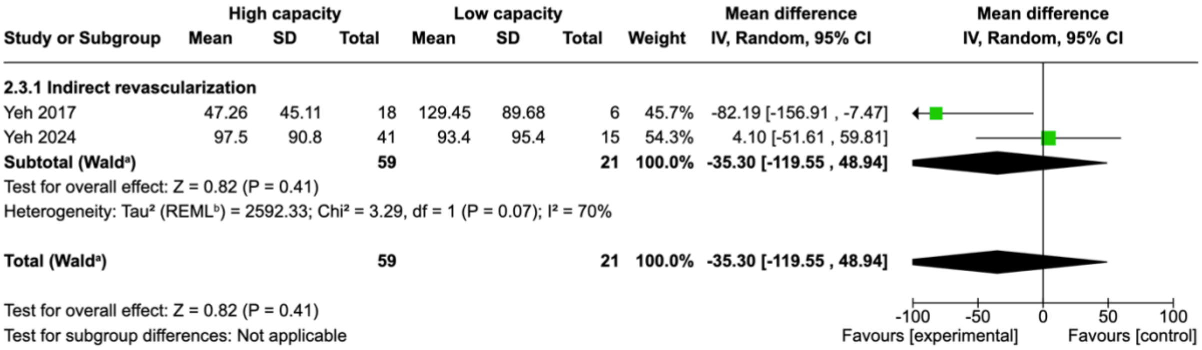

Test for overall effect: Z = 0.82 (P = 0.41)
Test for subgroup differences: Not applicable
Heterogeneity: Tau² (REML[b]) = 2592.33; Chi² = 3.29, df = 1 (P = 0.07); I² = 70%

Footnotes
[a]CI calculated by Wald-type method.
[b]Tau² calculated by Restricted Maximum-Likelihood method.

Supplementary Fig. 16 Forest plot showing that MCA PI is a non-significant predictor of high bypass capacity within a timeframe of 3 to 6 months. MCA – middle cerebral artery; PI – pulsatility index; IV – inverse variance; SD – standard deviation; CI – confidence interval.

	Experimental			Control				Mean difference
Study or Subgroup	Mean	SD	Total	Mean	SD	Total	Weight	IV, Random, 95% CI
**2.4.1 Indirect revascularization**								
Yeh 2017	0.71	0.2	18	0.68	0.31	6	18.0%	0.03 [-0.23 , 0.29]
Yeh 2024	0.69	0.18	41	0.67	0.22	15	82.0%	0.02 [-0.10 , 0.14]
**Subtotal (Wald[a])**			**59**			**21**	**100.0%**	**0.02 [-0.09 , 0.13]**
Test for overall effect: Z = 0.38 (P = 0.70)								
Heterogeneity: Tau^2 (REML[b]) = 0.00; Chi^2 = 0.00, df = 1 (P = 0.95); I^2 = 0%								
**Total (Wald[a])**			**59**			**21**	**100.0%**	**0.02 [-0.09 , 0.13]**

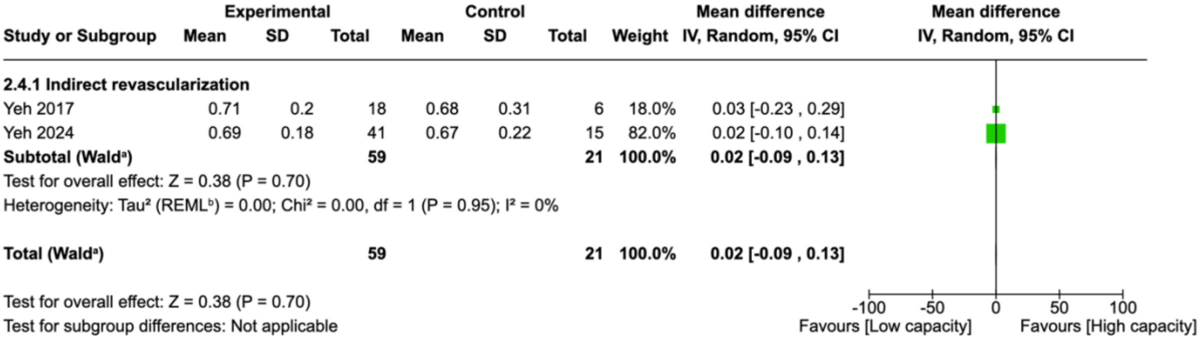

Test for overall effect: Z = 0.38 (P = 0.70)
Test for subgroup differences: Not applicable
Heterogeneity: Tau^2 (REML[b]) = 0.00; Chi^2 = 0.00, df = 1 (P = 0.95); I^2 = 0%

Footnotes
[a]CI calculated by Wald-type method.
[b]Tau^2 calculated by Restricted Maximum-Likelihood method.

Supplementary Fig. 17 Forest plot showing that PCA MFV is a non-significant predictor of high bypass capacity within a timeframe of 3 to 6 months. PCA – posterior cerebral artery; MFV – mean flow velocity; IV – inverse variance; SD – standard deviation; CI – confidence interval.

	High capacity			Low capacity				Mean difference
Study or Subgroup	Mean	SD	Total	Mean	SD	Total	Weight	IV, Random, 95% CI
**2.5.1 Indirect revascularization**								
Yeh 2017	119.27	43.53	18	86.49	35.3	6	43.0%	32.78 [-1.89 , 67.45]
Yeh 2024	113	37.2	41	116.9	36.9	15	57.0%	-3.90 [-25.77 , 17.97]
**Subtotal (Wald[a])**			**59**			**21**	**100.0%**	**11.87 [-23.72 , 47.46]**
Test for overall effect: Z = 0.65 (P = 0.51)								
Heterogeneity: Tau^2 (REML[b]) = 453.97; Chi^2 = 3.08, df = 1 (P = 0.08); I^2 = 67%								
**Total (Wald[a])**			**59**			**21**	**100.0%**	**11.87 [-23.72 , 47.46]**

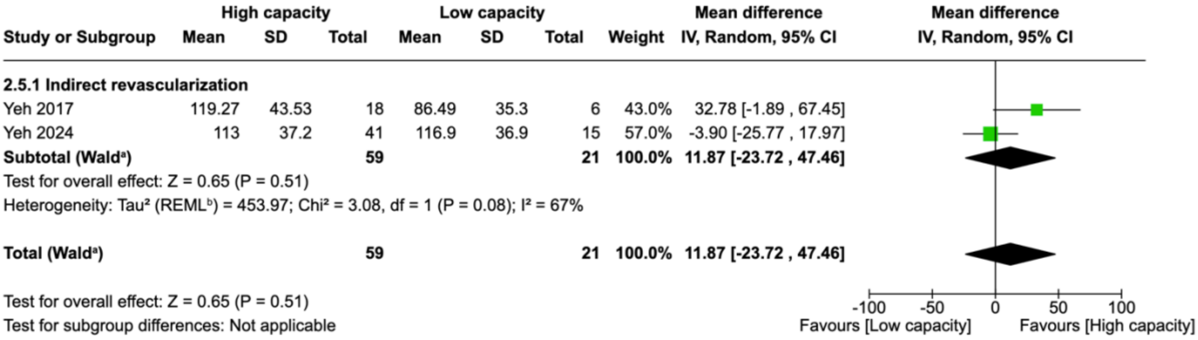

Test for overall effect: Z = 0.65 (P = 0.51)
Test for subgroup differences: Not applicable
Heterogeneity: Tau^2 (REML[b]) = 453.97; Chi^2 = 3.08, df = 1 (P = 0.08); I^2 = 67%

Footnotes
[a]CI calculated by Wald-type method.
[b]Tau^2 calculated by Restricted Maximum-Likelihood method.

Supplementary Fig. 18 Forest plot showing that PCA PI is a non-significant predictor of high bypass capacity within a timeframe of 3 to 6 months. PCA – posterior cerebral artery; PI – pulsatility index; IV – inverse variance; SD – standard deviation; CI – confidence interval.

	High capacity			Low capacity				Mean difference
**Study or Subgroup**	**Mean**	**SD**	**Total**	**Mean**	**SD**	**Total**	**Weight**	**IV, Random, 95% CI**
**2.13.1 Indirect revascularization**								
Yeh 2017	0.89	0.34	18	0.75	0.21	6	33.4%	0.14 [-0.09 , 0.37]
Yeh 2024	0.81	0.29	41	0.83	0.22	15	66.6%	-0.02 [-0.16 , 0.12]
**Subtotal (Wald[a])**			**59**			**21**	**100.0%**	**0.03 [-0.11 , 0.18]**
Test for overall effect: Z = 0.44 (P = 0.66)								
Heterogeneity: Tau² (REML[b]) = 0.00; Chi² = 1.34, df = 1 (P = 0.25); I² = 26%								
**Total (Wald[a])**			**59**			**21**	**100.0%**	**0.03 [-0.11 , 0.18]**

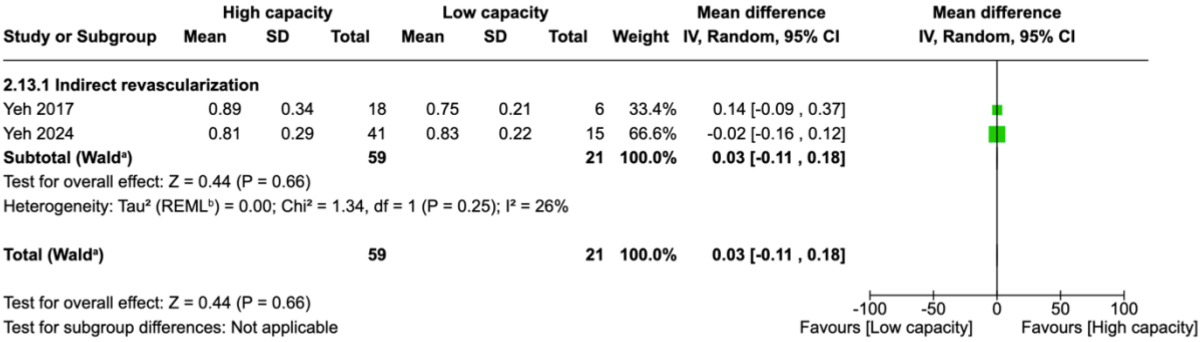

Test for overall effect: Z = 0.44 (P = 0.66)
Test for subgroup differences: Not applicable
Heterogeneity: Tau² (REML[b]) = 0.00; Chi² = 1.34, df = 1 (P = 0.25); I² = 26%

Footnotes

[a]CI calculated by Wald-type method.

[b]Tau² calculated by Restricted Maximum-Likelihood method.
